# Early childhood risk factors for rhinoconjunctivitis in adolescence: a prospective birth cohort study

**DOI:** 10.1186/s13601-017-0147-x

**Published:** 2017-04-03

**Authors:** Elisabeth Soegaard Christiansen, Henrik Fomsgaard Kjaer, Esben Eller, Carsten Bindslev-Jensen, Arne Høst, Charlotte Gotthard Mortz, Susanne Halken

**Affiliations:** 1 Department of Dermatology and Allergy Center, Odense Research Centre for Anaphylaxis (ORCA), Odense University Hospital, University of Southern Denmark, 5000 Odense C, Denmark; 2 Hans Christian Andersen Children’s Hospital, Odense University Hospital, University of Southern Denmark, Sdr. Boulevard 29, 5000 Odense C, Denmark

**Keywords:** Rhinoconjunctivitis, Risk factor, Adolescence, Logistic regression, Predictors, Birth cohort

## Abstract

**Background:**

Rhinoconjunctivitis is a global health problem and one of the most common chronic conditions in children. Development of rhinoconjunctivitis depends on both genetic and environmental factors. Many studies have investigated rhinoconjunctivitis, but only few studies have evaluated the risk factors for non-allergic rhinoconjunctivitis in children finding family history of atopic diseases and gender to be of importance. The aim of this study was to investigate possible risk factors in early life for rhinoconjunctivitis, allergic as well as non-allergic, in adolescence.

**Methods:**

The children in the Danish Allergy Research Center cohort were examined eight times from birth to 14 years of age. Visits included questionnaire-based interview, clinical examination, skin prick test and specific IgE. We used univariate and multivariate logistic regression to investigate the relationship between early-life risk factors and the development of rhinoconjunctivitis, allergic as well as non-allergic, in adolescence.

**Results:**

Follow-up rate at 14-years was 66.2%. The prevalence of rhinoconjunctivitis was 32.8%. Family history of atopic diseases (aOR 2.25), atopic dermatitis (aOR 3.24), food allergy (aOR 3.89), early sensitization to inhalant and food allergens (aOR 2.92 and aOR 3.13) and male gender (aOR 1.90) were associated with allergic rhinoconjunctivitis but not with non-allergic rhinoconjunctivitis. Early environmental tobacco exposure was inversely associated with rhinoconjunctivitis (aOR 0.42), allergic (aOR 0.47) as well as non-allergic (aOR 0.43).

**Conclusion:**

Different patterns of associations were revealed when stratifying rhinoconjunctivitis in allergic and non-allergic suggesting that allergic rhinoconjunctivitis and non-allergic-rhinoconjunctivitis are different phenotypes.

## Background

Rhinoconjunctivitis (RC) is a global health problem and one of the most common chronic conditions in children [1] with a prevalence that is still increasing [2]. When symptoms occur as a result of exposure to an allergen to which the individual is sensitized, the term allergic-rhinoconjunctivitis is used, but few longitudinal studies have used the combination of symptoms and measurement of sIgE [3].

Development of RC including allergic RC depends on both genetic and environmental factors [4]. Studies have indicated that gender [5, 6], family atopy [2, 4–6], early sensitization [4, 7, 8], food allergy [9] and atopic dermatitis [4, 9] are risk factors for subsequent development of rhinoconjunctivitis. The association between breastfeeding [2, 5], having pets [10], early exposure to tobacco smoke [2, 11, 12], social class [4], early wheeze [13], obesity [14], older sibling(s) [15–17] and allergic RC in adolescence is more contradictory.

Only a few studies have evaluated the prevalence [3, 18] and risk factors [17, 19] for non-allergic RC in children and they found family history of atopic diseases and gender to be of importance.

The Danish Allergy Research Center (DARC) cohort is a prospective birth cohort study with 6 follow-up examinations during the first 3 years of life and further two at 6 and 14 years. This gives a unique opportunity to explore risk factors in early childhood for the development of RC in adolescence. The aim of this study is to investigate possible risk factors in early life for RC, allergic as well as non-allergic, in adolescence.

## Methods

### Study population

The DARC cohort is a prospective non-interventional birth cohort study comprising 562 of 1095 consecutively enrolled full-term children born in the first two weeks of each month at Odense University Hospital, Denmark from November 1998 to November 1999. The children were evaluated during the first month of life and follow-up investigations were performed at 3, 6, 12, 18 months and 3, 6 and 14 years of age. All visits included questionnaire-based interviews, clinical examination, skin prick test (SPT), specific IgE (s-IgE), and at 6 and 14 years also spirometry. A detailed description was published previously [18].

### Diagnostic criteria

Rhinoconjunctivitis (RC) at 14 years was defined as: At least two separate episodes in the previous 12 months with one or more of the following symptoms: sneezing, runny, blocked nose, or itchy, red, watery eyes apart from a upper airway infection.

Allergic rhinoconjunctivitis (allergic RC) at 14 years was defined as having symptoms of RC and s-IgE ≥ 0.35 kU/l (ImmunoCAP, Thermo Fisher Scientific, Sweden) and/or a positive SPT (ALK-ABELLO, Copenhagen, Denmark) with a mean wheal diameter ≥3 mm larger than the negative control to at least one of the inhalant allergens: grass, birch, mugwort, horse, dog, cat, *Dermatophagoides pteronyssinus*, *Dermatophagoides farinae*, *Cladosporium herbarum* and *Alternaria alternata.*

Non-allergic rhinoconjunctivitis (non-allergic RC) at 14 years was defined as symptoms of RC and s-IgE ≤ 0.35 kU/l and a negative SPT.

Sensitization up to 3 years of age was measured by s-IgE ≥ 1.43 SU/ml, as analyzed with Magic Lite (ALK-ABELLÓ, Denmark) to the inhalant or food allergens (Table 1). Also a higher cut off value with s-IgE ≥ 4.0 SU/ml (Magic Lite) was investigated. S-IgE measured by Magic Lite ≥1.43 and ≥4.0 SU/ml corresponds to ImmunoCAP ≥0.35 and ≥0.70 kU/l, respectively [20].

**Table 1:** Potential risk factors for rhinoconjunctivitis (RC), allergic RC and non-allergic RC, at 14 years

Risk factors 0–3 years	Reference
Gender, boys	Girls
Family history of atopic diseases at baseline^a^ (FH)	No FH
Early sensitization to inhalant allergens^b^	No sensitization
Early sensitization to food allergens^c^	No sensitization
Food allergy^d^ (FA)	No FA
Atopic dermatitis^e^ (AD)	No AD
Early wheeze: at least two episodes of wheezing	No wheeze
Elevated cord blood IgE ≥ 0.3 kU/l (CB-IgE)	CB IgE < 0.3 kU/l
Daily exposure to tobacco smoke from parents or others in the household at more than two visits (ETS)	No ETS
Maternal tobacco smoking in pregnancy	No ETS in utero
Cat and/or dog keeping (pets)	No pets
Exclusive breastfeeding ≥3 months (eBF)	eBF ≤ 3 months
Social class^f^	High
Overweight at 3 years (BMI girls ≤17.6 and boys ≤17.8) [27]	No overweight
Cesarean section	No section
Older siblings (siblings)	No older siblings

^a^One of both parents with atopic disease

^b^Elevated s-IgE (Magic Lite, ALK Abello) for one or more of the inhalant allergens grass, birch, mugwort, horse, cat, dog, *Dermatophagoides pteronyssinus*, *Dermatophagoides farinae*, *Alternaria alternata* or *Cladosporium herbarum* antibodies at one or more visits up to 3 years

^c^Elevated s-IgE (Magic Lite, ALK Abello) for one or more of the food allergens cow’s milk, hen’s egg, wheat, peanut, codfish, shrimp or soy at one or more visits up to 3 years

^d^Food allergy (FA) was diagnosed by controlled elimination/challenge procedures with a positive oral food challenge (DBPCFC or OCFC) according to EAACI guidelines [28]

^e^Based on the Hanifin-Rajka criteria [29]

^f^Classified according to the grouping system of the Danish social research institute class 1–5 [30] 1–2 were considered “high”, 3–5 “low”

### Statistical methods

Risk factors are described and defined in Table 1.

Crude and adjusted effect estimates were analyzed with (multiple) logistic regression and given as crude odds ratio (cOR) and adjusted odds ratio (aOR) with 95% confidence interval (95% CI). *P* values ≤0.05 were considered significant.

Based on knowledge from the literature the variables gender, family history (FH), early sensitization, food allergy (FA) and atopic dermatitis (AD) were identified as risk factors [4, 5, 7, 9, 13] for developing RC and allergic RC and included in the multiple logistic regression model without further analysis. Gender [17] and FH [17, 19] were identified as risk factors for non-allergic RC and therefore included in this model without further analysis.

All other potential risk factors in Table 1 were tested individually with RC, allergic RC and non-allergic RC using univariate logistic regression analysis and included in the final model for RC, allergic RC and non-allergic RC if *P* value was <0.10.

Correlation between risk factors were tested using Spearman’s correlation coefficients and negligible correlation was found (r ≤ 0.3) except between the variables maternal smoke in pregnancy and exposure to tobacco smoke (ETS) where the correlation was moderate (r = 0.34). Therefore, all variables were retained for further analysis.

All analyses were performed using STATA/SE (Stata Corporation, College Station, TX, USA).

## Results

**Fig. 1:**
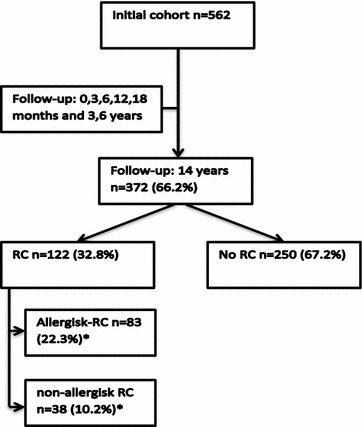
Flow-chart of the DARC cohort and the distribution of rhinoconjunctivitis (RC) in the 14 years follow-up investigation. Reference group was no RC. *One participant with RC had neither SPT nor IgE measured therefore could not be categorized as allergic or non-allergic

A total of 372 (66.2%) of the initial cohort (n = 562) completed the 14-years investigation, of whom 353 participated in at least 6 study visits. Flow-chart is presented in Fig. 1. Of the 190 not participating in the 14-years follow-up investigation significant less were in social class 1–2 and significantly more had mothers who smoked during pregnancy and at birth. A detailed description of the 14 years follow-up investigation was reported earlier [21].

At 14 years 32.8% (122/372) had RC, 22.3% (83/372) had allergic-RC and 10.2% (38/372) non-allergic-RC, the proportion of children with persistent symptoms were equally distributed in those having allergic-RC as well as non-allergic-RC but more with allergic-RC used antiallergic treatment (Table 2). One had neither SPT performed or s-IgE measured and therefore could not be classified as allergic or non-allergic (Fig. 1). A total of 46.8% (174/372) had FH, 54.8% (204/372) had early sensitization to foods, mostly to cow’s milk, egg and wheat, while 30.4% (113/372) had early sensitization to inhalant allergens. A total of 4.8% (18/372) had verified FA and 23.4% (87/372) had AD. As many as 29.6% (110/372) were exposed to tobacco smoke (ETS) during pregnancy and 51.6% (192/372) up to the age of 3 (Table 3).

**Table 2:** Symptoms score and use of medication in those with symptoms of allergic and non-allergic RC

	Allergic RC % (n)	Non-allergic RC % (n)
Allergic symptoms at least 1 h daily for at least 2 weeks from eyes and nose	37.4 (31)	36.8 (14)
Use of oral antihistamines	12.1 (52)	23.7 (9)
Use of nasal sodium cromoglicate	27.7 (23)	5.2 (2)
Use of intranasal corticosteroids	25.3 (21)	10.5 (4)
Use of other antiallergic medication	10.8 (9)	5.2 (2)
Use of any of the above medication	68.7 (57)	39.5 (15)

**Table 3:** Crude effect estimates for the associations between risk factor in early childhood and RC, allergic RC and non-allergic RC at 14 years of age

	RC			Allergic RC			Non-allergic RC		
	Total^a^	Yes/no	cOR (CI 95%)	Total^a^	Yes/no	cOR (CI 95%)	Total^a^	Yes/no	cOR (CI 95%)
Gender			1.53 (0.99–2.36)			2.33 (1.39–3.91)*			0.65 (0.32–1.33)
Boys (n = 178)		67/111		165	54/111		124	13/111	
Girls (n = 194)		55/139		168	29/149		164	25/139	
FH (n = 174)	372	72/102	2.09 (1.35–3.25)*	333	52/102	2.43 (1.46–4.06)*	288	19/102	1.45 (0.73–2.88)
Early sensitization to inhalant allergens (n = 113)	372	47/66	1.75 (1.10–2.77)*	333	37/66	2.24 (1.34–3.76)*	288	10/66	1.00 (0.46–2.16)
Early sensitization to foods (n = 204)	372	80/124	1.94 (1.24–3.03)*	333	56/124	2.11 (1.25–3.55)*	288	24/124	1.74 (0.86–3.52)
s-IgE > 4.00 SU/ml to inhalant allergens (n = 56)	372	31/25	3.07 (1.72–5.48)*	333	28/25	4.58 (2.48–8.47)*	288		
s-IgE > 4.00 SU/ml to food allergens (n = 57)	372	34/23	3.81 (2.12–6.84)*	333	30/23	5.59 (3.01–10.39)*	288		
FA (n = 18)	372	14/4	7.97 (2.57–24.78)*	333	12/4	10.39 (3.25–36.22)*	288	2/4	3.42 (0.60–19.33)
AD (n = 87)	372	48/39	3.51 (2.13–5.78)*	333	41/39	5.28 (3.05–9.15)*	288	7/39	1.22 (0.50–2.97)
Early wheeze (n = 59)	372	23/36	1.38 (0.78–2.45)	333	17/36	1.53 (0.81–2.90)	288	6/36	1.12 (0.44–2.86)
CB IgE 0.3 (n = 62)	301	22/40	1.11 (0.62–2.00)	268	18/40	1.44 (0.76–2.73)	232	3/40	0.41 (0.12–1.43)
ETS (n = 192)	372	47/145	0.45 (0.29–0.71)*	333	34/145	0.50 (0.30–0.83)*	288	13/145	0.38 (0.18–0.77)*
Maternal tobacco smoking in pregnancy (n = 110)	372	30/80	0.69 (0.42–1.13)	333	24/80	0.86 (0.50–1.49)*	288	6/80	0.40 (0.16–0.99)*
Breast feed (n = 247)	362	78/169	0.76 (0.48–1.21)	323	51/169	0.67 (0.40–1.13)	277	26/169	0.99 (0.46–2.11)
Pets (n = 231)	372	75/156	0.96 (0.62–1.50)	333	52/156	1.01 (0.61–1.69)	288	22/156	0.83 (0.41–1.66)
Social class (n = 305)	372	99/206	0.92 (0.53–1.61)	333	64/206	0.72 (0.39–1.32)	288	34/206	1.82 (0.61–5.38)
Overweight at 3 years (n = 25)	320	8/17	0.86 (0.36–2.07)	285	3/17	0.46 (0.13–1.60)	243	5/17	1.87 (0.64–5.45)
Cesarean section (n = 44)	365	18/26	1.53 (0.80–2.92)	327	11/26	1.38 (0.65–2.93)	285	7/26	1.92 (0.77–4.79)
Older siblings (n = 182)	372	51/131	0.65 (0.42–1.01)	333	32/131	0.57 (0.34–0.94)*	288	19/131	0.91 (0.46–1.80)

^a^Total number of children with data included in the analysis

* *P* ≤ 0.05

Crude odds estimates for all risk factors are giving in Table 3.

### RC and allergic RC

FH and AD up to 3 years of age were associated with RC and allergic RC in adolescence as well as early sensitization to inhalant and food allergens with s-IgE ≥ 4.00 SU/ml (Magic Lite corresponding to 0.7 kU/l by ImmunoCap). ETS was associated with a lower prevalence of RC and allergic RC (Table 4).

**Table 4:** Adjusted effect estimates (aOR CI 95%) for associations with RC, allergic RC and non-allergic RC at 14 years of age

	RC	RC inc. s-IgE ≥ 4.00 SU/ml	Allergic RC	Allergic RC inc s-IgE ≥ 4.00 SU/ml	Non-allergic RC
	aOR (CI 95%)	aOR (CI 95%)	aOR (CI 95%)	aOR (CI 95%)	aOR (CI 95%)
Gender	1.26 (0.78–2.04)	1.30 (0.80–2.12)	1.90 (1.06–3.41)*	2.08 (1.14–3.81)*	0.64 (0.31–1.32)
FH	2.01 (1.25–3.23)*	2.01 (1.24–3.26)*	2.25 (1.27–3.99)*	2.32 (1.28–4.18)*	1.45 (0.72–2.92)
Early sensitization to inhalant allergens	1.31 (0.78–2.20)		1.65 (0.91–3.02)		NI
Early sensitization to foods	1.62 (0.99–2.67)*		1.57 (0.86–2.87)		NI
s-IgE ≥ 4.00 SU/ml to inhalant allergens		2.03 (1.06–3.90)*		2.92 (1.43–5.96)*	NI
s-IgE ≥ 4.00 SU/ml to food allergens		2.38 (1.15–4.93)*		3.13 (1.41–6.95)*	NI
FA	3.49 (1.00–12.16)	2.36 (0.62–8.92)	3.89 (1.08–14.08)*	2.08 (0.52–8.31)	NI
AD	2.44 (1.37–4.33)*	2.11 (1.17–3.81)*	3.24 (1.72–6.12)*	2.68 (1.38–5.19)*	NI
ETS	0.42 (0.26–0.69)*	0.42 (0.26–0.69)*	0.47 (0.26–0.83)*	0.48 (0.27–0.87)*	0.43 (0.20–0.92)*
Maternal tobacco smoking in pregnancy	NI	NI	NI	NI	0.56 (0.21–1.46)
Older siblings	0.64 (0.40–1.03)	0.66 (0.41–1.07)	0.53 (0.30–0.94)*	0.55 (0.31–1.00)	

*NI* not included due to univariate logistic regression analysis

* *P* ≤ 0.05

We found no association between early wheeze, maternal smoking in pregnancy, pets, breastfeeding, elevated CB-IgE, social class, overweight at 3 years, cesarean section and RC and allergic RC in adolescence.

An association between male gender, FA and sibling were only found in those with allergic RC (Table 4).

### Non-allergic RC

Among the 38 children with non-allergic RC no association with FH, early sensitization to inhalant and food allergens, FA, and AD was found, while ETS was significantly associated with less non-allergic RC in adolescence (Table 4).

## Discussion

In this study, different patterns of associations were revealed by stratifying RC in allergic and non-allergic. It appears that atopic heredity and early atopic manifestations are important for the development of allergic RC in adolescence but less important for the development of non-allergic RC. Furthermore, early ETS was inversely associated with RC (aOR 0.42), allergic-RC (aOR 0.47) and non-allergic RC (aOR 0.43), in adolescence.

Family atopy (FH) was associated with allergic RC (aOR 2.25). The relation between FH and the development of rhinitis are well established in cohort studies [2, 4, 5, 9, 19]. Some argue that maternal atopy are more important than paternal atopy [22], but in the MAAS (Manchester Asthma and Allergy Study) study [2] both maternal asthma (OR 2.38) and paternal hayfever (OR 1.96) were significantly associated with RC at age 5. Having two parents with allergy was associated with allergic rhinitis at 13 years in the MAS (Multi-center Allergy Study) study [5] (OR 3.1) as was parental isolated hay fever with allergic rhinitis at 8 years in the Swedish BAMSE (Children Allergy Milieu Stockholm study) study [19] (OR 2.2), whereas parental isolated asthma or eczema was not. Our study did not have the sufficient power to differentiate between the different parental atopic diseases.

Food allergy (FA) was associated with allergic RC in adolescence (aOR 3.89). Among those participating at 14 years of age, 54.8% were sensitized to food allergens early but only 4.8% had verified FA up to 3 years, which may reflect the transient and often benign nature of food sensitization in infancy [8]. The association between food allergy in infancy and RC in adolescence is less investigated. In the Isle of Wight cohort an increased risk of nasal symptoms in those with FA at 1 and 4 years was found in the univariate analysis but this association disappeared in the multivariate analysis [9]. Using a higher cut-off value both early sensitization to inhalant allergens and foods reach significance, while the association between FA and allergic RC disappeared. This probably reflects sensitization in those with FA. Thus, weak sensitization to food and inhalant allergens in early life might be less important as a risk factor for subsequent development of allergic RC.

No association was found between FH, early sensitization to inhalant allergens or foods, AD and FA and non-allergic RC. Though analyzed in another way (time-to-event analysis) the same patterns were found in the German MAS study [7] while other studies of non-allergic RC found an association between FH and non-allergic RC [17, 19]. An explanation of the different patterns of association seen in the two diseases might be that allergic RC and non-allergic RC are different phenotypes with non-allergic RC not being driven by atopy but environmental factors. Another explanation might be that the children with non-allergic RC had milder symptoms and therefore may outgrow RC.

AD was in our study associated with both RC (aOR 2.44) and allergic-RC (aOR 3.24). Of 87 children that had AD up to 3 years, 48 developed RC in adolescence. Thus these children might follow a trajectory of the atopic march, whereas in two English cohort studies MAAS and ALSPAC (Avon Longitudinal Study of Parents and Children) [23] only a small proportion of children followed trajectory profiles similar to the atopic march.

In our study boys had a higher risk of allergic-RC in adolescence (aOR 1.90). A consistent male predominance in the prevalence of allergic rhinitis was seen in all ages in the German MAS cohort but only in those with allergic parents [5]. In the Isle of Wight cohort a male predominance was seen at 18 years in those with allergic rhinitis [6] while there was female predominance in non-allergic-rhinitis. Though not reaching statistical significance a tendency to female predominance in those with non-allergic RC was found in our study.

We found that having older siblings were inversely associated with allergic-RC (aOR 0.53). The relation between large family size and less hayfever was described by Strachan in 1989 [16] and summarized in the “hygiene hypothesis” [16] suggesting that allergic diseases were prevented by infections in early childhood transmitted by contact with older siblings. Other cohort studies report similar associations, the German MAS cohort [5] for allergic rhinitis and the Tucson cohort [24] for asthma. Though studies of the association between having older siblings and the development of RC are fairly consistent in the conclusions of the possible beneficial effect [5, 16] we found such an association for allergic RC, but not for non-allergic RC.

ETS before the age of 3 was inversely associated with RC (aOR 0.42), allergic-RC (aOR 0.47) and non-allergic RC (aOR 0.43) in adolescence. The same tendency was seen between maternal smoking during pregnancy and non-allergic RC though not reaching statistical significance. The inverse association between ETS and RC, allergic as well as non-allergic RC could be due to bias in different ways, firstly as report-bias, secondly that parents with atopy smoked less than parents without atopy and thirdly as a disease-related modification of exposure [25] if parents to symptomatic children quit smoking. Firstly, all follow-up investigations included measurement of parental expiratory carbon monoxide as an objective measurement of smoking which could enhance parents to report smoking more accurately. Secondly, ETS was reported in 51.6%, and 29.6% of the mothers smoked during pregnancy. Parental atopy at baseline was found in 46.8 and 46% of those reported smoking. Thirdly, parents to those developing allergic diseases are prone to stop smoking and among parents to children with atopic dermatitis and/or wheeze before 3 years only 24% were smokers at the 14 years follow-up. This indicates a possible reverse causation and that not only smoking exposure in early life, but also the long-lasting exposure was important. Another Danish study [12] found the same tendency with an inverse association for prenatal smoke exposure OR 0.8 and hayfever in adolescence and a large ISAAC study [11] found an inconsistent or weak association between ETS and RC. An explanation of a possible beneficial effect of ETS on RC could be an immunomodulatory effects of nicotine, which also indicates a possible beneficial effect of smoking in some inflammatory and neurodegenerative diseases [26].

### Strengths and limitations

Our study has several limitations. First of all our study population are relative small and since the study included only 51.3% of those fulfilling the inclusion criteria, there is a risk for selection bias. However, this group did not differ from those that declined participation originally [18]. Participants at 14 years belonged to a higher social class and were less exposed to maternal smoking during pregnancy and birth, which may influence the prevalence of atopic diseases. Besides, participants in a cohort study may have increased awareness of atopic diseases resulting in an overestimation of the prevalence and on the other hand in avoidance of possible risk factors, which may result in lowering the prevalence. Despite of this we found a high prevalence of RC in adolescence which was in line with finding of other cohorts [6, 23]. In our study we used symptoms within the last 12 months and diagnosis of RC by a doctor and sensitization was determined both by SPT and measurement of s-IgE.

Of the 372 children participating at 14 years of age, 353 attended at least 6 follow-up investigations. The DARC cohort had 6 study visits in the first 3 years which allow us to compensate for missing data from 1 or more visits. Most of the environmental risk factors were only included if these were present at two visits or more, e.g. ETS, to validate the outcome.

## Conclusion

In this study we used a widely used model to estimate the association between early-life risk factors and RC. Different patterns of association were revealed when stratifying RC in allergic and non-allergic suggesting that allergic RC and non-allergic RC are different phenotypes. Early sensitization is a risk factor for developing allergic RC in adolescence and a higher cut-off value of s-IgE seems associated with a higher risk.

Non-allergic RC is not driven by atopic heredity or early atopic manifestations but possibly by other mechanism to intrinsic or environmental factors.

In the future it appears important to explore the nature and clinical characteristics and course of non-allergic-RC in order to find optimal treatment options.

Furthermore the pattern of early sensitization to specific allergens and the relation to later atopic diseases seem important to investigate focusing on possible preventive measures.

## Abbreviations

AD: atopic dermatitis
Allergic-RC: allergic rhinoconjunctivitis
aOR: adjusted odds ratio
BMI: body mass index
CB IgE: cord blood IgE
cOR: crude odds ratio
DARC: The Danish Allergy Research Center
eBF: exclusive breastfeeding
ETS: environmental tobacco smoke
FA: food allergy
FH: family history
Nonallergic-RC: nonallergic rhinoconjuntivitis
RC: rhinoconjunctivitis
S-IgE: specific IgE
SPT: skin prick test
